# S-1 induced discoid lupus erythematosus-like lesions and long-term complete response for para-aortic lymph node recurrence of pancreatic ductal adenocarcinoma: a case report

**DOI:** 10.1186/s40792-018-0460-1

**Published:** 2018-06-07

**Authors:** Ryosuke Yamaga, Koji Tezuka, Shuichiro Sugawara, Toshihiro Watanabe, Ichiro Hirai, Tamio Suzuki, Wataru Kimura

**Affiliations:** 1 First Department of Surgery, Graduate School of Medical Science, Yamagata University, 2-2-2 Iida-Nishi, Yamagata, 990-9585 Japan; 2 Department of Dermatology, Graduate School of Medical Science, Yamagata University, Yamagata, Japan

**Keywords:** DLE, S-1, Chemotherapy, Skin eruption, Pancreatic cancer, Long-term survival

## Abstract

**Background:**

Metastatic recurrence after resection of pancreatic cancer is considered to be an incurable disease, and discoid lupus erythematosus (DLE)-like lesions are known as a side effect of fluorouracil agents. We report a very rare case of metastatic recurrence of pancreatic cancer in a Japanese man with DLE-like lesions in which long-term complete response was achieved through S-1 monotherapy.

**Case presentation:**

A 65-year-old man who had undergone distal pancreatectomy with splenectomy for pancreatic body cancer and had received adjuvant gemcitabine developed postoperative para-aortic lymph node recurrence 17 months after surgery. S-1 monotherapy was started. About 2 weeks after starting this treatment, he developed an erythematous rash on the face and scalp. DLE was diagnosed by skin biopsy. The eruptions were aggravated by the administration of S-1 and improved during periods of respite from S-1. Yet as CA19-9 was reduced by almost half 1 month after starting S-1 chemotherapy, S-1 chemotherapy was continued at a reduced dose. CA19-9 decreased to within a normal range within 6 months after starting S-1 chemotherapy, and a reduction in lymph node metastasis was detected through imaging. The patient is still alive without recurrence or metastasis 113 months after surgery.

**Conclusions:**

Even in patients with S-1-induced DLE-like lesions, continuation of S-1 is possible if the dose and duration of S-1 are appropriately regulated and medical therapy is administered for the skin lesions. Further investigation into the possible correlation between skin rash and clinical benefit in connection with S-1 is strongly warranted.

## Background

S-1 is an oral fluoropyrimidine drug which contains tegafur, a prodrug of 5-fluorouracil (FU), 5-chloro-2,4-dihydroxypyridine (CDHP), and potassium oxonate (Oxo). The anticancer activity of tegafur is enhanced by CDHP and its gastrointestinal toxicity is reduced by Oxo [1]. Through advances in surgical techniques [2] and adjuvant chemotherapy [3], the prognosis of pancreatic cancer after surgical resection is improving, but the prognosis of recurrent pancreatic cancer is still poor [4, 5].

Discoid lupus erythematosus (DLE)-like eruptions have been reported as a side effect of FU agents such as UFT and capecitabine [6–9], but there are no reported cases of DLE-like lesions induced by S-1 in the English literature.

We report a rare case in which S-1 monotherapy induced DLE-like lesions but also yielded long-term complete response.

## Case presentation

**Fig. 1:**
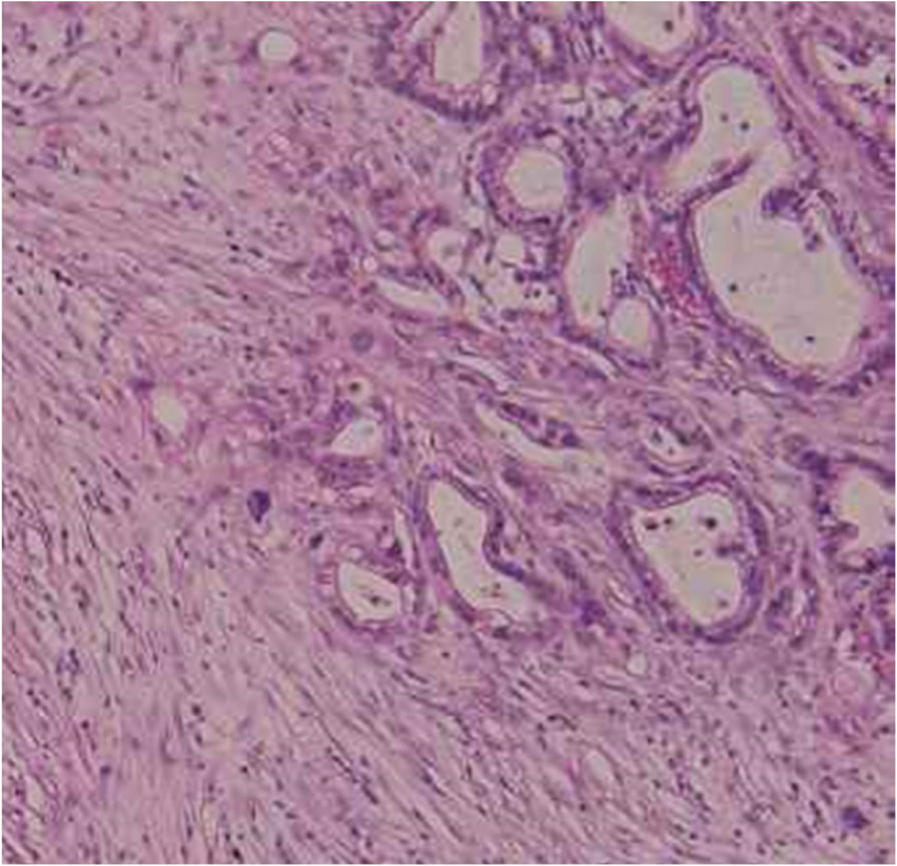
Histopathological findings of resected specimen of the pancreas. Moderately to poorly differentiated ductal adenocarcinoma was seen in the pancreas

A 65-year-old man underwent distal pancreatectomy with splenectomy for pancreatic body cancer. The pathological stage was Stage IIB (T3, N1, M0) according to the American Joint Committee on Cancer (AJCC) classification [10]. Two of the 30 dissected lymph nodes were positive for metastasis. Histopathological diagnosis based on the resected specimens was moderately to poorly differentiated ductal adenocarcinoma (Fig. 1). Perineural and vascular invasion were observed.

The patient had undergone appendectomy for appendicitis at 13 years of age. He had been treated with antihypertensives from the age of 55. Additionally, he had received a diagnosis of diabetes mellitus during a preoperative examination of the pancreatic cancer, and insulin therapy was started at 3 months postoperation.

At his preoperative examination, carcinoembryonic antigen (CEA) was within a normal range (2.51 mg/dl), but carbohydrate antigen 19-9 (CA19-9) was elevated (126.8 U/ml).

**Fig. 2:**
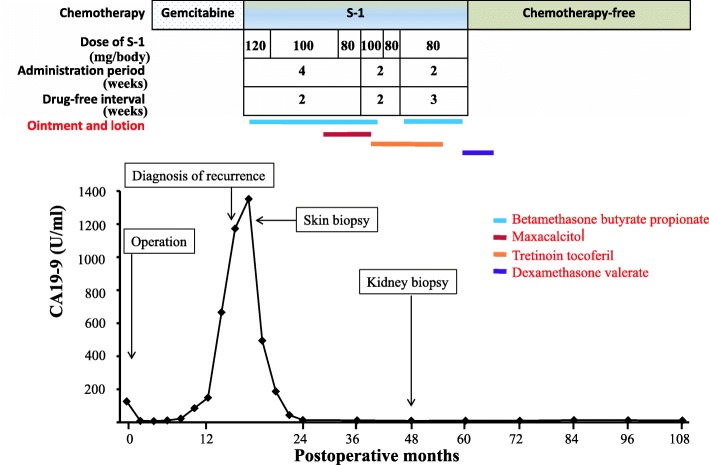
Clinical course, postoperative changes of CA19-9, and dose and schedule of S-1 chemotherapy

**Fig. 3:**
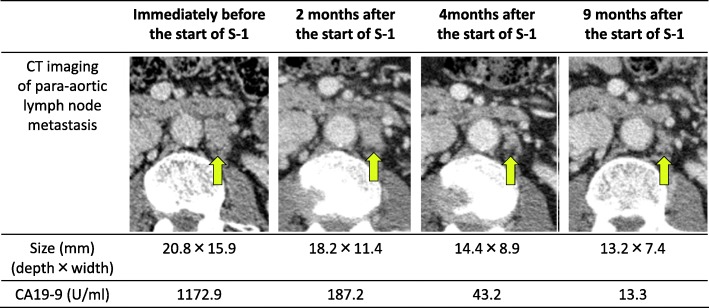
Changes in the appearance and size of para-aortic lymph node metastasis on axial CT imaging and CA19-9 after the start of S-1

Although adjuvant chemotherapy with gemcitabine was administered, levels of the tumor marker CA19-9 began to increase from 12 months after the operation (Fig.2), and computed tomography (CT) showed a para-aortic lymph node metastasis at 17 months after the operation (Fig.3). At the time of his diagnosis with recurrence, CA19-9 had increased to 1172.9 U/ml.

**Fig. 4:**
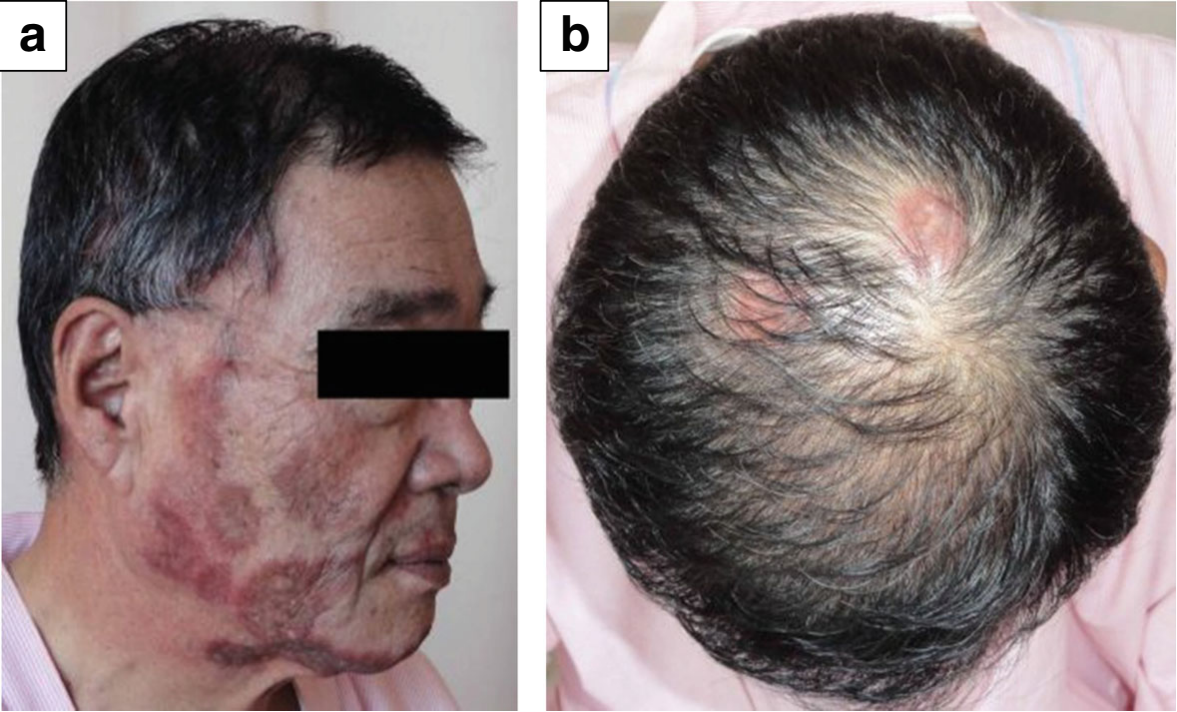
Erythematous lesions on the face (**a**) and scalp (**b**)

**Fig. 5:**
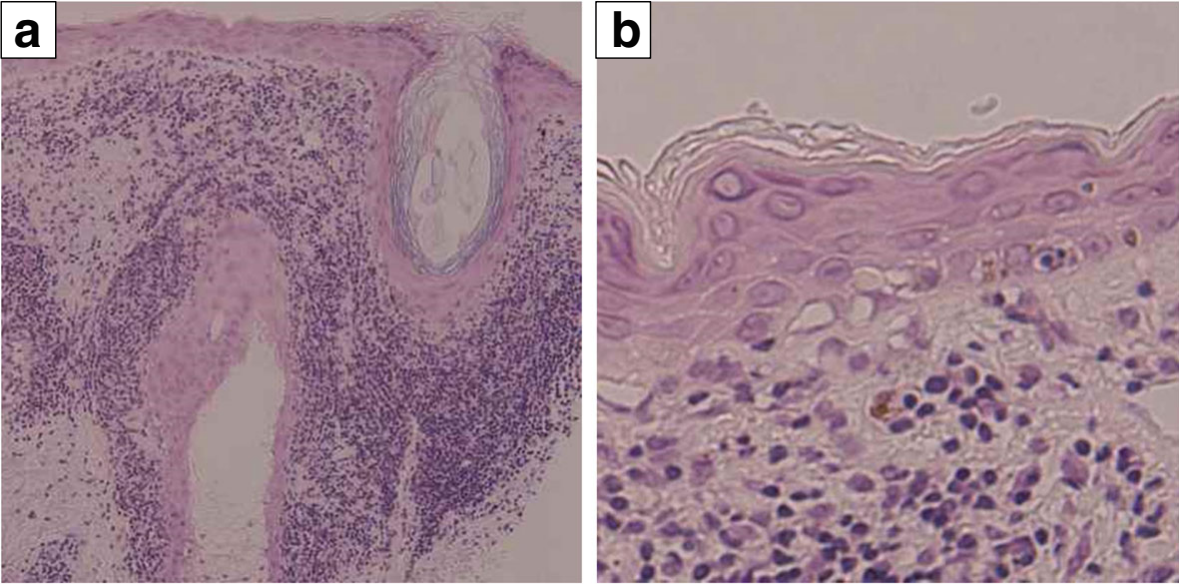
Pathological findings in an erythematous eruption on the right cheek. **a** Infiltration of inflammatory cells was seen around the skin appendages (HE, × 100). **b** Liquefaction degeneration of the basement cells and epidermal atrophy were seen (HE, × 200)

Subsequently, S-1 monotherapy (120 mg/day) was administered as first-line chemotherapy for recurrent pancreatic cancer. About 2 weeks after starting S-1, however, the patient developed an erythematous eruption with itching on sun-exposed portions of the skin such as the face, scalp, and precordium (Fig. 4a, b). A skin biopsy specimen taken from the right cheek showed liquefaction degeneration of the basement cells, epidermal atrophy, and infiltration of inflammatory cells, which mainly consisted of lymphocytes around the skin appendages (Fig. 5a, b). These elements were suggestive of discoid lupus erythematosus eruption.

At the onset of this eruption, laboratory test results showed elevated IgG (1740 mg/dl), IgA (431 mg/dl), blood cell sedimentation rate (29 mm/h), and complement level (46.5 U/ml). C3, C4, antinuclear antibody, anti-single-stranded DNA antibody, anti-double-stranded DNA antibody, anti-SS-A/Ro antibody, anti-SS-B/La antibody, anti-Sm antibody, and anti-RNP antibody were all negative.

Lesions notwithstanding, the patient’s CA19-9 level was reduced by almost half in the first month after starting S-1 chemotherapy (120 mg/day). Therefore, S-1 chemotherapy was continued at a reduced dose of 100 mg/day after discussions with the patient and a dermatologist. The eruptions were treated with topical ointment and lotion (betamethasone butyrate propionate, maxacalcitol, tretinoin tocoferil, and dexamethasone valerate) (Fig. 2). The eruptions were aggravated by the administration of S-1 and improved within 2 weeks after the discontinuation of S-1. The pigmentation associated with the eruption gradually spread during periods of S-1 administration. We continued S-1 chemotherapy for 41 months after having adjusted the dose and dosage interval (Fig. 2). The subjective and objective symptoms improved when the rest interval was changed to 2 weeks followed by 3 weeks of treatment and the dose was reduced to 80 mg/day.

CA19-9 decreased to within a normal range (< 37.0 U/ml) within 6 months of starting S-1 chemotherapy and did not increase again (Fig. 2). At the time of the patient’s diagnosis with recurrence, enhanced CT revealed lymph node metastasis with a slowly growing minor axis of 15.9 mm on the left side of the aorta (Fig. 3). Within 9 months of starting S-1 chemotherapy, however, the mass had reduced to 7.4 mm (Fig. 3). As a result, we considered the patient to exhibit complete response (CR) in accordance with the Response Evaluation Criteria In Solid Tumors (RECIST) guidelines [11].

**Fig. 6:**
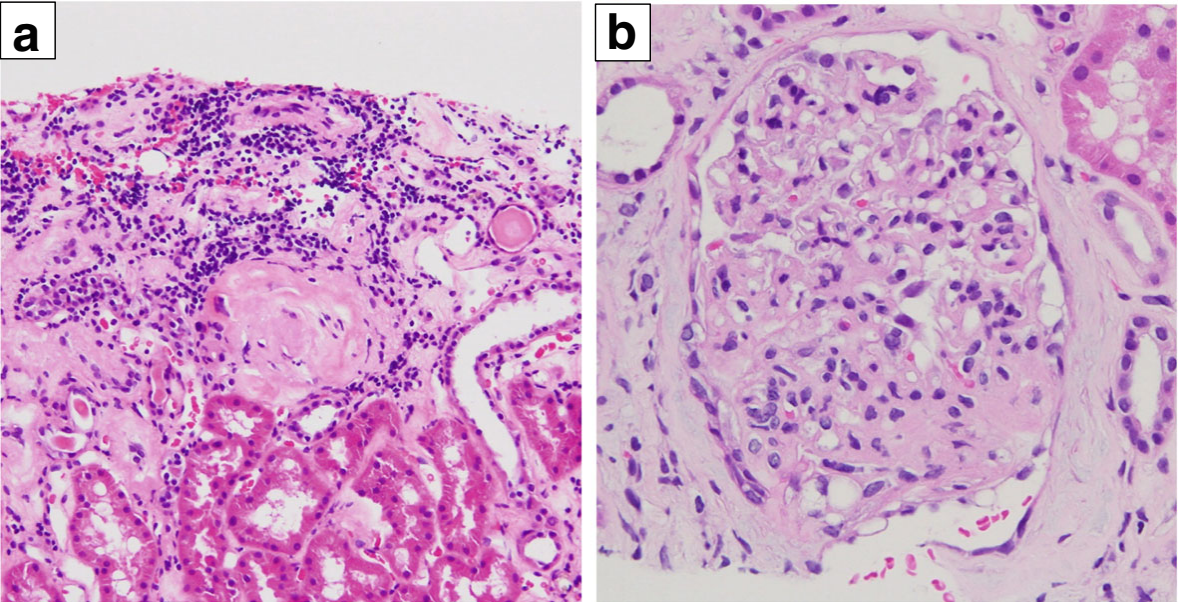
Light microscopy findings of kidney biopsy. **a** Light microscopy showed nodular expansion of mesangium and infiltration of lymphocytes into the interstitium (HE, × 100). **b** Light microscopy of glomeruli showed increased mesangial matrix and increased mesangial cellularity (HE, × 200)

Proteinuria, which had been observed in a random urine examination prior to the administration of S-1, increased over a period of 30 months from the start of S-1 chemotherapy. Laboratory data also revealed hypoalbuminemia (3.1 g/dl). A kidney biopsy showed mesangial proliferative glomerulonephritis (Fig.6a, b). Given the patient’s medical history, the nephritis was thought to have been caused by hypertension; accordingly, we continued to administer S-1 chemotherapy while also taking steps to manage blood pressure.

The patient’s S-1 chemotherapy was completed in 41 months. The patient is alive without recurrence or metastasis 113 months after surgery (96 months after diagnosis of recurrence and 53 months after termination of S-1). The regions that were affected by erythema during S-1 chemotherapy have now developed alopecia, but their pigmentation is improved.

### Discussion

Drug eruptions induced by FU agents are classified into keratotic vesicular, pigmented type, photosensitivity type, DLE-like eruption type, erythematous papule type, lichen planus type, and others [6]. Of all skin eruptions induced by FU agents, DLE-like eruptions account for almost 10% [6]. Drug-induced DLE-like lesions are skin eruptions resembling DLE that are caused by continuous drug exposure and resolved after discontinuation of the offending drug [9, 12]. The clinical presentation of DLE consists of well-demarcated discoid erythematous macules or papules on sun-exposed areas such as the scalp, face, ears, and neck, which gradually develop into an indurated discoid plaque with an adherent scale. Atrophy and pigmentation of the plaque and scarring alopecia occur in the chronic course [12]. The present case likewise had permanent alopecia in the areas affected by eruptions.

DLE is included in the category of chronic cutaneous lupus erythematosus [12]. The clinical course of drug-induced DLE-like eruptions is different from that of idiopathic DLE in that it can be improved by the discontinuation of the offending drug [6, 9, 12].

The cause of DLE-like eruptions is believed to be ultraviolet light exposure to basal cells that have been damaged by FU agents and have thereby become highly photosensitive [6]. Additionally, an autoimmune mechanism is suspected to be involved in DLE-like eruptions because many autoantibody-positive cases exhibit DLE-like eruptions [8].

To our knowledge, this is the first report of DLE-like lesions induced by S-1 in the English literature, though there have been several reports in the Japanese literature [13, 14]. In these reports, the mean duration of S-1 administration before eruption development was almost 50 days, and the mean total dose before eruption development was 3.8 g [13, 14]. In our case, the duration of S-1 administration was shorter at 14 days, and the total dose was lower at 1.68 g.

Discontinuation of the offending drug, topical steroid therapy, and protection from light are likely to be effective in treating DLE-like eruptions, but in some reported cases, eruptions have been improved by topical steroid therapy and protection from light without discontinuation of S-1 [13, 14]. In our case, because S-1 was highly effective, we continued the administration of S-1 while minimizing lesion symptoms by regulating the dosing period and the dose while treating the lesions with corticosteroid ointment.

For patients with pancreatic cancer with distant metastases, prognosis remains poor [4, 5]. The partial response rate of S-1 monotherapy for locally advanced and/or metastatic pancreatic cancer is reported to range from 4 to 21%, but the CR rate is reportedly 0% [4, 5, 15]. There have been several case reports of CR in metastatic pancreatic cancer by S-1 monotherapy alone [16–18], but no cases of long-term CR more than 4 years after termination of S-1 have been reported; in this regard, the present case is quite rare. It has been suggested that the autoimmune response might also contribute to tumor reduction [19]. The antiribosomal P autoantibodies are detectable in 12 to 16% of patients with systemic lupus erythematosus and can inhibit the growth of pancreatic cancer cells, in vitro and in vivo [19]. There is a possibility that DLE-like lesions and long-term CR coexist because there is no evidence that patient with DLE-like lesions are more sensitive to 5-FU, including S-1, and there are several case reports of CR in metastatic pancreatic cancer by S-1 monotherapy without DLE-like lesions [16–18]. Due to its rarity, we suspect that activation of an immune function might have been one of the factors in our patient’s long-term CR. The antiribosomal P antibodies should have been examined at the onset of DLE to determine the contribution of immunological activation. In keeping with this hypothesis, correlations between skin rash and clinical benefit have been reported in connection with anti-epidermal growth factor receptor (EGFR) antibody, tyrosine kinase inhibitors, and capecitabine [20–22]. Further investigation into the possibility of a correlation between skin rash and clinical benefit in connection with S-1 is strongly warranted.

It should be noted that our patient had a diagnosis of mesangial proliferative glomerulonephritis by kidney biopsy during S-1 chemotherapy. Because he had exhibited hypertension and proteinuria prior to S-1 chemotherapy, we considered his hypertension to be the cause of his glomerulonephritis, but we cannot rule out the possibility that the administration of S-1 was a factor in his increased proteinuria given that persistent proteinuria and worsening of renal function has been reported in idiopathic DLE [23]. Therefore, the proteinuria and renal function of patients with DLE-like drug eruptions should be carefully examined at regular intervals during administration of S-1.

Our case suggests that, with attentive follow-up and appropriate regulation of the dosing period and the dose, using topical steroid therapy and protection from light could be a promising treatment option for patients with DLE-like drug eruptions while the clinical response offered by the anticancer drug is obtained. On the other hand, because exacerbation of the skin damage induced by S-1 may interfere with daily life and may become a predisposing risk factor for the development of squamous cell carcinoma [24], detailed discussion of these matters with patients is important.

## Conclusions

We report on a rare case in which complete response was achieved by continued S-1 administration after the occurrence of discoid lupus erythematosus-like lesions.

## Abbreviations

AJCC: American Joint Committee on Cancer
CA19-9: Carbohydrate antigen 19-9
CDHP: 5-Chloro-2,4-dihydroxypyridine
CEA: Carcinoembryonic antigen
CR: Complete response
CT: Computed tomography
DLE: Discoid lupus erythematosus
EGFR: Anti-epidermal growth factor receptor
FU: Fluorouracil
Oxo: Oxonate
RECIST: Response Evaluation Criteria in Solid Tumors
